# Transsaccadic visual perception of foveal compared to peripheral environmental changes

**DOI:** 10.1167/jov.21.6.12

**Published:** 2021-06-23

**Authors:** Sonia Bansal, Wilsaan M. Joiner

**Affiliations:** 1 Department of Neuroscience, George Mason University, Fairfax, VA, USA; 2 Maryland Psychiatric Research Center, Department of Psychiatry, University of Maryland School of Medicine, Baltimore, MD, USA; 3 Department of Bioengineering, George Mason University, Fairfax, VA, USA; 4 Department of Neurobiology, Physiology and Behavior, University of California Davis, Davis, CA, USA; 5 Department of Neurology, University of California Davis, Davis, CA, USA

**Keywords:** corollary discharge, saccade, fovea, peripheral, visual perception

## Abstract

The maintenance of stable visual perception across eye movements is hypothesized to be aided by extraretinal information (e.g., corollary discharge [CD]). Previous studies have focused on the benefits of this information for perception at the fovea. However, there is little information on the extent that CD benefits peripheral visual perception. Here we systematically examined the extent that CD supports the ability to perceive transsaccadic changes at the fovea compared to peripheral changes. Human subjects made saccades to targets positioned at different amplitudes (4° or 8°) and directions (rightward or upward). On each trial there was a reference point located either at (fovea) or 4° away (periphery) from the target. During the saccade the target and reference disappeared and, after a blank period, the reference reappeared at a shifted location. Subjects reported the perceived shift direction, and we determined the perceptual threshold for detection and estimate of the reference location. We also simulated the detection and location if subjects solely relied on the visual error of the shifted reference experienced after the saccade. The comparison of the reference location under these two conditions showed that overall the perceptual estimate was approximately 53% more accurate and 30% less variable than estimates based solely on visual information at the fovea. These values for peripheral shifts were consistently lower than that at the fovea: 34% more accurate and 9% less variable. Overall, the results suggest that CD information does support stable visual perception in the periphery, but is consistently less beneficial compared to the fovea.

## Introduction

To efficiently collect visual information from the environment, saccadic eye movements rapidly shift the location of the fovea several times per second. The disruption to visual input accompanying saccades creates a complex problem for the visual system: (1) during a saccade visual sampling is suppressed until the eyes land and fixation occurs and (2) after each saccade the image of the visual world shifts across the retina because of the eye movement. Despite the shifts of the retinal image with each saccade, our brain creates a seamless and visually stable percept of the world by actively and accurately integrating visual and motor information over multiple eye movements (e.g., Hall & Colby, 2011; Melcher & Colby, 2008; Wurtz, 2008; Wurtz, Joiner, & Berman, 2011).

There are various complementary theories on how the brain compensates for movement-induced disruptions to visual perception and maintains spatial and temporal constancy across saccades: comparison of the scene before and after the saccade to determine a mismatch (Sperry, 1950; von Holst & Mittelstaedt, 1950), calibration of the visual environment based on reference objects (Deubel, Bridgeman, & Schneider, 1998; Deubel, Schneider, & Bridgeman, 2002), construction of spatiotopic reference frames for visual memory (Burr & Morrone, 2011; Zimmermann, Morrone, & Burr, 2013a; Zimmermann, Morrone, Fink, & Burr, 2013b; Zimmermann, Morrone, & Burr, 2014), and predictive remapping of objects during the eye movement (Jonikaitis, Szinte, Rolfs, & Cavanagh, 2013; Rolfs, Jonikaitis, Deubel, & Cavanagh, 2011; Szinte, Carrasco, Cavanagh, & Rolfs, 2015). Most experimental studies and subsequent theories are based on the ability to detect a change in the environment that occurs during the eye movement. For example, humans accurately perceive small (<1°) transsaccadic displacements to saccade targets, both when the target is blanked before the displacement and when the target shift is not proceeded by target blanking (horizontal movements: Bridgeman, Hendry, & Stark, 1975; Collins, Rolfs, Deubel, & Cavanagh, 2009; Deubel, Schneider, & Bridgeman, 1996; Deubel et al., 1998; Deubel, Schneider, & Bridgeman, 2002; Niemeier, Crawford, & Tweed, 2003; Niemeier, Crawford, & Tweed, 2007; Ostendorf et al. 2010; Joiner, Cavanaugh, FitzGibbon, & Wurtz, 2013a; Joiner, Cavanaugh, & Wurtz, 2013b; Wexler & Collins, 2014; vertical movements: Bansal, Jayet Bray, Peterson, & Joiner, 2015; two-dimensional extent: Jayet-Bray, Bansal, & Joiner, 2016).

The neural mechanisms underlying all these theories of perceptual stability necessitate knowledge about the upcoming saccade movement vector and updating a map in retinotopic coordinates (Duhamel, Colby, & Goldberg, 1992; Melcher and Colby, 2008; Nakamura & Colby, 2002; Sommer & Wurtz, 2006; Wurtz, 2008). Considering that several areas involved in saccadic remapping are also involved in oculomotor control, it is likely the visual system utilizes outgoing signals (i.e., internally generated motor signals) during spatial remapping (Cavanaugh, Berman, Joiner, & Wurtz, 2016). Supported by psychophysical and neurophysiology studies in nonhuman primates (Joiner et al., 2013a; Joiner et al, 2013b; Sommer & Wurtz, 2002; Sommer & Wurtz, 2004a; Sommer & Wurtz, 2004b) and humans (Bellebaum, Daum, Koch, Schwarz, & Hoffmann, 2005; Bellebaum, Hoffmann, Koch, Schwarz, & Daum, 2006; Bridgeman et al.,1975; Duhamel et al., 1992; Joiner et al., 2010; Joiner et al., 2013a; Ostendorf et al. 2010), this remapping is hypothesized to be mediated by an internal corollary discharge (CD) signal. CD signals, found to occur across the animal kingdom and in several areas of the motor system (Crapse & Sommer, 2008), do not generate movements but are copies of the efferent motor command sent to the muscles to produce movement (Sperry, 1950; von Holst & Mittelstaedt, 1950). When combined with internal computations of visual processing, corollary discharge signals allow the brain to predict the impending effects and consequences of the saccade, thereby selecting the appropriate retinal and spatial remapping across cortical cells on the basis of the saccade movement vectors (Sommer & Wurtz, 2006; Sommer & Wurtz, 2008).

Based on this framework, when we view an object, the visual system integrates and compares presaccadic and postsaccadic retinal images by taking into account the presaccadic scene and CD to predict the postsaccadic scene. The CD internally relays the information of the impending saccade vector (movement amplitude and direction) to the required neural sensory areas to compensate for the impending trans-saccadic changes in the retinal position attributable to the upcoming eye movement (Duhamel et al., 1992; Gottlieb, Kusunoki, & Goldberg, 1998; Hall & Colby, 2011; Nakamura & Colby, 2002; Sommer & Wurtz, 2006). We (Bansal et al., 2015; Jayet-Bray et al., 2016) and others (Bridgeman et al., 1975; Collins et al., 2009; Li & Matin, 1990a, 1997; Niemeier et al., 2007; Ostendorf et al., 2010; Joiner et al., 2010) have confirmed that humans are sensitive to transsaccadic displacements despite the variability of the saccadic end points, and that this sensitivity scales with movement amplitude such that compensation for saccade-induced disruptions to visual input has an uncertainty proportional to the magnitude of the motor signal. Furthermore, it has been shown that perisaccadic mislocalization is stronger for vertical saccades (Grujic, Brehm, Gloge, Zhuo, & Hafed, 2018) and CD-driven compensation for an initial saccade by a second saccade is less accurate for vertical saccades (Joiner et al., 2010). Thus there is increasing but isolated knowledge on how the metrics of the saccade influence the purported ability of the CD to compensate for saccade-induced visual input disruptions.

Despite the evidence summarized above, the majority of previous studies have largely focused on transsaccadic visual changes occurring at the fovea. Subsequently, there is limited knowledge on how extraretinal information (e.g., corollary discharge) possibly benefits perception when trans-saccadic displacements occur in the periphery. Therefore the complete spatial extent of the CD-based compensation remains unclear. In one of the few studies examining the benefits of extraretinal information in this manner, Li & Matin (1997) conducted a series of experiments in which three observers judged whether a test flash, presented either after a saccade or during a period of fixation, was displaced to the left or right of a reference point viewed earlier. Relative contributions of extraretinal and retinal factors to saccadic suppression of displacement (SSD) were investigated by measuring displacement thresholds. The experiments, involving saccades ranging from 4° to 12° in length, separated effects of saccade size from the effects of retinal eccentricity of the reference point, and also separated the effects of retinal eccentricity of the test flash from both. The authors found that ∼20% of the total influence on SSD was derived from retinal influences of the test flash and reference point; 80% was attributed to the extraretinal influence accompanying saccade size. However, in their experiments, the authors only studied horizontal eye movements, and it is not clear how the CD representation for saccade metrics (amplitude and direction) influences visual perception at the periphery for vertical environmental changes.

Previously we quantified the extent corollary discharge/extraretinal signals contribute to perception at the fovea compared to solely relying on visual error (Jayet-Bray et al., 2016; Bansal et al., 2018). We showed that overall the perceptual estimate was approximately 50% more accurate and 35% less variable than estimates based solely on this visual information in healthy control subjects (Jayet-Bray et al., 2016). In addition, we showed that schizophrenia patients, hypothesized to have a disruption in the CD mechanism, relied more on the experienced visual error and consequently underestimated the target position compared to control subjects (Bansal et al., 2018). However, it is largely unknown how this CD (extraretinal) signal may contribute to perception of peripheral changes compared to relying only on visual error information. This information would assist in forming a more complete picture of how the stability of the entire visual scene is maintained across saccades, not just at the fovea. Thus, as an extension of our previous study, here we examined transsaccadic visual perception at the fovea and periphery for different movement amplitudes (4° and 8°) and directions (horizontal and vertical). Our experimental design allowed a systematic examination of (1) visual perception of environmental scene changes at the same spatial location across different saccade amplitudes (i.e., the location of the environmental change remained the same, but occurred either in the periphery or at the fovea based on the required saccade amplitude) and (2) how perception of peripheral changes is modified by saccade amplitude (i.e., the saccade amplitude changed, but the environmental change was always a set distance from the movement goal).

## Methods

### Participants

Twelve healthy human subjects (seven males and five females, 18–25 years of age) were recruited for this study. Participants reported normal or corrected-to-normal vision and were unaware of the purpose of the study, which consisted of making perceptual judgments about the location of presented visual stimuli. Subjects did not obtain any training in the tasks before collecting experimental data and received payment after completing two test sessions. Experimental protocols were approved by the Institutional Review Board of George Mason University, and informed consent was obtained from each participant.

### Apparatus and measurement

Eye movements were recorded using the Eyelink II eye tracker (head-mounted binocular eye tracker, 500 Hz temporal resolution, 0.2° spatial resolution; SR Research Ltd., Mississauga, ON, Canada). Stimuli were presented on a 19-in CRT-monitor (screen resolution 1024 × 768 pixels; refresh rate 110 Hz) at a viewing distance of 62.5 cm. The fixation cross was white with a luminance of 56 cd/m^2^ viewed against a black background of luminance of less than 0.1 cd/m^2^. The initial saccadic target was an orange (RGB values of 255, 102, 0) circle (diameter of 0.5°). The reference point was a white (255,255,255) circle (diameter of 0.5°), presented simultaneously with the saccadic target either with an overlap of (0.07°) over the saccadic target, or at the peripheral location (4°) away from the saccadic target.

Subjects were seated in a dimly lit room in a stationary chair with their head stabilized by a chinrest. Stimulus presentation, eye movement and manual keyboard response data acquisition were achieved using real-time experimental control software (Experiment Builder, SR Research Ltd.). At the start of each experiment session, a nine-point gaze calibration was performed followed by a nine-point validation.

### Task and procedure

**Figure 1.**
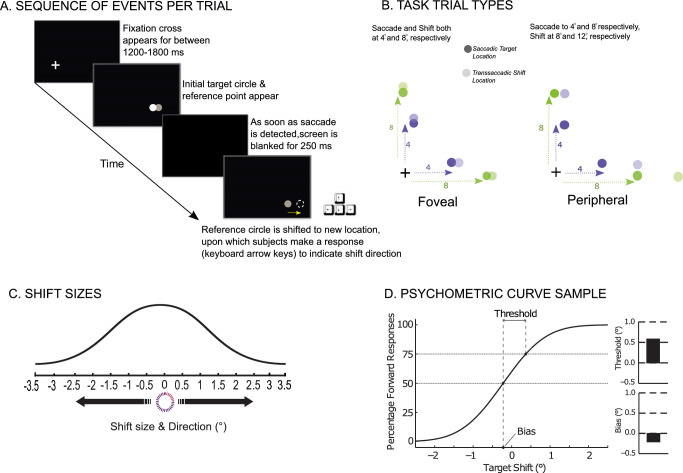
Transsaccadic shift detection task. (A) Sequence of events per trial. Each trial began with a fixation cross (0.3° in extent) that was offset such that it was 11.5° from the left and bottom screen edge respectively. Subjects were required to maintain fixation on this cross for a variable period (random duration between 1200 and 1800 ms). After the fixation period, an initial target was presented at either one of two amplitudes (4° or 8°) and two directions (upward or rightward) from the fixation cross—a total of four possible locations. Subjects were required to make a saccadic eye movement toward this initial target. Once the eye position exceeded a virtual square window (3.2° in width, ±1.6° from fixation) around the fixation point, the cross and target were extinguished and followed by a 250-ms blank period. The reference point then reappeared at a shifted randomized position between ±3.5° (See panel C). After the reappearance of the reference point, the subject made a manual response. (B) Task trial types. The display consisted of a saccadic eye-movement target at the mentioned locations, and a reference point for perceptual discrimination (the dark and lighter symbols, respectively). For trials in which perceptual discrimination of transsaccadic shifts occurred at the fovea, the reference point and the saccadic eye-movement target partially overlapped at the same location. For trials in which this discrimination occurred in the periphery, the reference point was 4° away (further to the right or further upward) from the initial saccadic eye-movement target. This design resulted in four trial types: (i) Saccade to the 4° target, reference point/shift at 4° (foveal change); (ii) Saccade to the 4° target, reference point/shift at 8° (peripheral change); (iii) Saccade to the 8° target, reference point/shift also at 8° (foveal change) and (iv) Saccade to the 8° target, reference point/shift at 12° (peripheral change). (C) Shift sizes. The reference point reappeared at a shifted randomized position between ±3.5 (0.5° increments, drawn from a Gaussian distribution centered at 0°, with the smaller, less prominent shifts being sampled more than the larger, more detectable shifts). (D) Psychometric function. Frequency of forward responses on the y-axis is plotted as a function of target displacement on the x-axis. These manual response data were fitted to a cumulative Gaussian distribution to determine two perceptual measures: the perceptual threshold and bias. The threshold is computed as the difference in reference point displacement between the 50% and 75% points of the psychometric curve. The perceptual bias is the perceptual null location and was taken as the displacement from zero at the point where the percentages of forward and backward responses were equal to 50%.

Figure 1 displays the task sequence and trial types. Each trial began with a fixation cross (0.3° in extent) that was offset such that it was 11.5° from the left and bottom screen edge respectively. Subjects were required to maintain fixation on this cross for a variable period (random duration between 1200 and 1800 ms; Figure 1A). After the fixation period and extinguishing of the cross, an initial target was presented at one of two amplitudes (4° or 8°) and two directions (upward or rightward) from the fixation cross—a total of four possible locations (Figure 1B). Subjects were required to make a saccade to the orange circular saccadic target once it appeared, along with the white reference point and were instructed to make a manual response to indicate whether the white circle had shifted left or right from its original location for horizontal locations, and upward or downward for vertical locations. The display consisted of a saccadic eye-movement target at the mentioned locations, and a reference point for perceptual discrimination (the dark and lighter symbols, respectively). For trials in which perceptual discrimination of transsaccadic shifts occurred at the fovea, the reference point and the saccadic eye-movement target overlapped at the same location (Figure 1B). For trials in which this discrimination occurred in the periphery, the reference point was 4° away (further to the right or further upwards) from the initial saccadic eye-movement target. This design resulted in four trial types: (i) Saccade to the 4° target, reference point/shift at 4° (4° foveal); (ii) Saccade to the 4° target, reference point/shift at 8° (4° peripheral); (iii) Saccade to the 8° target, reference point/shift also at 8° (8° foveal); and (iv) Saccade to the 8° target, reference point/shift at 12° (4° peripheral). Once the eye position exceeded a virtual square window (3.2° in width, ±1.6° from fixation) around the fixation point, the cross and target were extinguished and followed by a 250-ms blank period (Deubel et al., 1996). The reference point then reappeared at a shifted randomized position between ±3.5° (0.5° increments). The reference point shift was randomly drawn from a Gaussian distribution centered at 0°, with the smaller, less prominent shifts being sampled more than the larger, more detectable shifts (Figure 1A). After the reappearance of the reference point, the subject made a manual response, using keyboard arrow keys to indicate the direction in which the reference point shifted (upward/downward or leftward/rightward) and the shifted reference point was extinguished, and trial ended as soon as the manual response was made. Subjects had to make this manual response within 3000 ms but were given no instructions on reaction time. (All subjects responded within this allotted time period.) No feedback was given to subjects regarding response accuracy. For each direction (rightward or upward), amplitude (4° or 8°) and trial type (foveal or peripheral), 122 (randomized) trials were presented for a total of 976 (8 conditions × 122) trials spread equally over two study sessions.

### Saccade measures

Horizontal and vertical movements of both eyes were recorded, and the resulting data were visualized, filtered, and analyzed off-line with the MATLAB v 8.1.0 environment (MathWorks, Natick, MA, USA). During the task, saccade initiation was detected when the eye position exited the 3.2° square fixation window. For off-line analyses, as defined in previous studies by our group (Joiner & Shelhamer, 2006; Zorn, Joiner, Lasker, & Shelhamer, 2007; Bansal et al., 2015; Jayet-Bray et al., 2016) an eye movement was classified as a saccade if both eye velocity and acceleration exceeded 50°/s and 2000°/s^2^, respectively. Saccadic end points were examined for every amplitude and direction. Only trials in which (1) the saccade was initiated within the fixation window, (2) its distance exceeded one third of the initial target amplitude, and 3) the primary saccade end point was within the average eye position ± 2 standard deviations (SDs) were analyzed. On average 86.3% ± 2.1% of trials for each subject were included in the analysis.

### Analyses

#### Psychometric curves

Consistent with our previous work (Joiner et al., 2013a; Joiner et al., 2013b; Bansal et al., 2015; Bansal et al., 2018; Jayet-Bray et al., 2016), we derived postsaccadic estimates of the initial reference point location and quantified the difficulty in detecting reference shifts. These measures were based on psychometric curves (cumulative Gaussians) that were fitted to the proportion of manual responses and specified the relationship between the probability of forward responses and the magnitude of the reference shift. Psychometric functions from manual responses are hypothesized to be based on the experienced visual error (VE) and the CD of the saccadic eye-movement, because both types of information (the postsaccadic visual error and extraretinal information, such as the CD signal) are available to make perceptual judgments. Perceptual bias, inferred as the post-saccadic estimation of the location of the presaccadic (initial) reference point, was quantified as a shift from 0 at the point where the percentage of forward responses was 50% (Figure 1C). CD provides information about saccade metrics (hypo or hyper), and therefore this is purportedly used to make a perceptual estimation about the presaccadic reference point location with respect to the eye movement; this estimation is quantified by the bias measure. A positive bias indicated that the initial reference point location was estimated to be ahead of its actual position; a negative bias indicated that initial reference point location was estimated to be behind the actual position. (Note that when there is a lack of CD use, saccade endpoint errors could be used for this perceptual decision; the postsaccadic errors resulting from hypometric saccades would appear forward of the saccade end point more frequently. Thus more frequent forward reports would shift the psychometric function to the left, resulting in a negative bias.) As done previously (Bansal et al., 2015; Bansal et al., 2018; Jayet-Bray et al., 2016), we quantified the difference between the perceptual estimate and actual reference point location as a percent error of the reference location: bias divided by the initial reference point location scaled by 100. This was done to compare over- or underestimation of the initial reference point location to the percent gain of the initial saccade amplitude.

Perceptual threshold, calculated as difference in shift size between the 50% and 75% points on the psychometric curve, quantified perceptual sensitivity in detecting reference point shifts (Figure 1C); larger thresholds represent increased difficulty in perceiving trans-saccadic shifts. To determine the extent there was a systematic relationship between the saccade amplitude and the perceptual threshold, we compared the normalized thresholds. For each subject, we scaled each threshold by the mean saccade amplitude, defining the threshold as a percentage of movement length.

Further, as done in previous work (Bansal et al., 2018, Jayet-Bray et al., 2016) we derived hypothetical psychometric functions if the perceptual decision was driven by only the VE. In this case, we hypothesized that VE represents shifted reference point direction, and that the CD-based (or other extraretinal derived information) estimate of eye position is not used. On every trial we determined the difference between the eye position at the time of the reference point reappearance and the shifted reference location (VE). The direction of the resultant error vector was used as the basis for the simulated reference point shift judgment. The percentage of these VE-based forward judgments was plotted as a function of reference shift to obtain a hypothetical psychometric function. Our assumption in the VE-based cases is that subjects have no extraretinal/CD-based information about the saccade metrics; only post-saccadic VE information is available for the perceptual judgment. As such, here the decision is based on the error the subject experiences when the target is shown at the displaced location. Therefore, it is not the actual choice of the subject, but the choice that would be made based on the error experienced. This is a simplification, but provides a baseline under experimental conditions to determine how actual perceptual performance (using extraretinal information, specifically CD) is superior to the limited VE-based situation.

In summary, to compare the saccadic CD-related benefit for each of the aforementioned trial types, we derived measures of threshold and bias from psychometric curves, and related normalized thresholds and percent error of reference point location. We compared VE+CD to VE-only based threshold and percent error measures for each trial type as well.

In a novel analysis, we also examined whether the location of the reference point shift (foveal versus peripheral condition) had an effect on the time required to make a perceptual judgement. We derived manual reaction times per condition, separated by the size of the transsaccadic shift. The manual reaction time is derived as the time (in milliseconds) between reappearance of the reference point stimulus at the shifted location and button press time.

### Statistical analyses

The experimental group data across subjects were not significantly different from a normal distribution (Shapiro-Wilk test, *p* > 0.05). Statistical significance of multiple effects such as target amplitude (4° or 8°), saccade direction (rightward or upward), and shift location (foveal or peripheral) on perceptual measures was determined by two- or three-way analyses of variance (ANOVAs). To determine significant differences, post-hoc two-tailed *t*-tests and paired *t*-tests were used to compare results to set values (e.g., comparing variables to zero) or between conditions (e.g., VE-only based versus CD+VE-based). Unless otherwise noted, parametric assumptions were met for all statistical tests. For all tests the significance level was 0.05. Statistical analyses were performed using MATLAB and JASP software (JASP Team, 2020).

## Results

### Saccade and response measures

Table 1 summarizes the saccade metrics that we compared between shift location conditions (foveal vs. peripheral). For the 4° saccadic targets, even though saccades were slightly greater in amplitude when the environmental change was in the periphery versus the fovea, the effect of reference point location was not significant (F_1,11_ = 3.16, *p* = 0.10, η^2^_p_ = 0.22). Additionally, there was no significant difference in amplitude between saccade directions (horizontal versus vertical) (F_1,11_ = 2.35, *p* = 0.15, η^2^_p_ = 0.18) and the shift location by direction interaction effect was not significant (F_1,11_ < 0.001, *p* = 0.98, η^2^_p_ = 0.00). Likewise, for 8° saccadic eye-movement targets, none of these effects on saccade amplitude were significant: shift location (F_1,11_ = 2.94, *p* = 0.12, η^2^_p_ = 0.21), direction (F_1,11_ = 4.2, *p* = 0.07, η^2^_p_ = 0.28) and shift location by direction interaction effect (F_1,11_ = 0.79, *p* = 0.39, η^2^_p_ = 0.07). Thus, for both 4° and 8° saccadic eye-movement targets, saccade amplitudes were similar across movement directions, and reference shifts locations. Saccadic eye-movement latencies did not differ by direction (F_1,11_ = 0.44, *p* = 0.52, η^2^_p_ = 0.00) or reference shift location (F_1,11_ = 0.55, p = 0.47, η^2^_p_=0.00), but were longer for 8° saccades (F_1,11_ = 19.22, p = 0.001, η^2^_p_ = 0.39). Mean manual response reaction times were derived for each trial type. Manual response reaction times were greater for upward versus rightward targets (F_1,11_ = 27.81, *p* < 0.001, η^2^_p_ = 0.272), shorter for 4° than for 8° targets (F_1,11_ = 6.75, *p* = 0.025, η^2^_p_ = 0.38) and shorter for foveal versus peripheral shift detection (F_1,11_ = 31.02, *p* < 0.001, η^2^_p_ = 0.74). These differences in reaction time will be further addressed below.

**Table 1.** Saccade and manual response measures. *Notes*: Values are presented as mean (SD) for each measure. RT = reaction time; ms = milliseconds.

	Rightward		Upward	
	4°	8°	4°	8°
Saccade Amplitude, °				
Foveal	3.28 (0.36)	6.75 (0.28)	3.51 (0.61)	6.96 (0.56)
Peripheral	3.46 (0.35)	6.82 (0.36)	3.69 (0.53)	7.15 (0.48)
*P* value, paired *t*-test (Foveal vs. Peripheral)	0.06	0.18	0.33	0.08
Saccade Latency, ms				
Foveal	222.21 (27.63)	279.1 (31.76)	242.98 (20.01)	298.49 (38.58)
Peripheral	235.77 (31.89)	294.04 (36.56)	260.84 (37.28)	309.46 (55.38)
*P* value, paired *t*-test (Foveal vs. Peripheral)	0.11	0.46	0.24	0.27
Manual Response RT, ms				
Foveal	514.31 (94.11)	547.5 (114.2)	594.65 (124.61)	603.02 (128.29)
Peripheral	551.49 (114.75)	572.65 (116.92)	637.17 (131.59)	647.85 (124.09)
*P* value, paired *t*-test (Foveal vs. Peripheral)	0.048*	0.03*	0.02*	0.02*

### Perceptual performance

**Figure 2.**
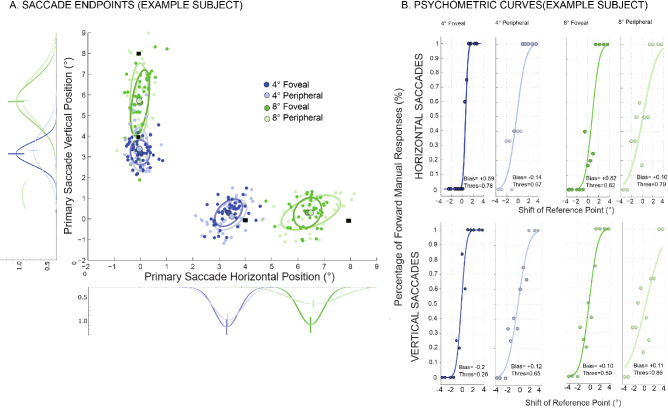
Perceptual performance and primary saccadic eye-movement offset positions for one subject. (A) Primary saccade variability. The primary saccade horizontal and vertical positions, along with the mean saccade amplitude (larger circle at center of ellipses), are displayed. Colored filled circles represent the end points for individual saccades, and the ellipses represent 95% confidence intervals for each target amplitude (blue: 4° saccadic eye-movement target, and green: 8° saccadic eye-movement target; lighter shading: reference location shift in the periphery; darker shading: foveal shift of reference location) Solid black squares indicate the target location. The traces to the left and below the plot are the respective distributions of the saccade end points, with the means indicated as vertical lines. (B) Perceptual performance measures. For each direction (horizontal, upper panel and vertical, lower panel), four psychometric functions (blue: 4°, green: 8°, lighter shading, shift in the periphery; darker shading, foveal shift) are shown with their respective perceptual bias and perceptual threshold measurements.

We examined the perceptual detection of trans-saccadic changes in the environment as subjects made eye movements to targets that required saccades of various amplitudes (4° and 8°) and directions (upwards and rightwards). With our novel design, we investigated this detection when the reference point shift occurred at the fovea (at the saccadic eye-movement target) versus the periphery (4° away from the saccadic eye-movement target). The primary saccade endpoints during this target displacement detection task for a sample subject are presented in Figure 2A. The colored filled small circles are the endpoints for individual saccades and the ellipses represent 95% confidence intervals around these endpoints for each amplitude and direction. The larger filled circles represent the mean saccade endpoints and the solid black squares represent the saccadic eye-movement target. Darker shading represents the foveal shift conditions, while the lighter shading represents shifts occurring in the periphery (see Methods). Consistent with previous reports, saccade variability (e.g. Abrams et al. 1989; van Beers 2007; Leigh & Zee, 2015) and target undershoot scaled with saccade amplitude (e.g., Collewijn et al. 1988; de Bie et al. 1987; Leigh & Zee, 2015); both the size of the ellipses and the distance between the colored squares and respective circles scale with movement amplitude.

The psychometric curves and corresponding measurements for the same sample subject are shown in Figure 2B. The functions are derived from the percentage of reported forward responses for the target-shift direction (see Methods). For both saccade directions, the slope of the psychometric functions largely decreased with the increase in movement amplitude, corresponding to an increase in the perceptual thresholds (Figure 2B). Thus the subject had increased difficulty distinguishing the direction of environmental change because the change occurred at a further eccentricity away from the fixation cross presented at the beginning of the trial (i.e. increased difficulty for longer saccadic target amplitudes, as well as shifts occurring in the periphery). Additionally, for this subject, the bias (the postsaccadic reference point shift that resulted in 50% forward responses) was largely positive (six of the eight cases) for both amplitudes, directions, and reference shift locations, indicating a mismatch between the estimated postsaccadic and presaccadic reference point location. Specifically, the positive bias demonstrates that the initial reference point location was perceived to be further away (rightwards or upwards) of its actual position. Thus these positive bias estimates are compatible with a CD signal whose gain was <1. Consistent with previous literature (e.g., Bridgeman et al., 1991 [passive eye rotation]; Grüsser, Krizic, & Weiss, 1987 [visual afterimages]; Szinte & Cavanagh, 2011 and Zimmermann et al., 2018 [transsaccadic apparent motion perception]), subjects expected more movement undershoot of the reference location than they observed postsaccadically.

### Perceptual thresholds

**Figure 3.**
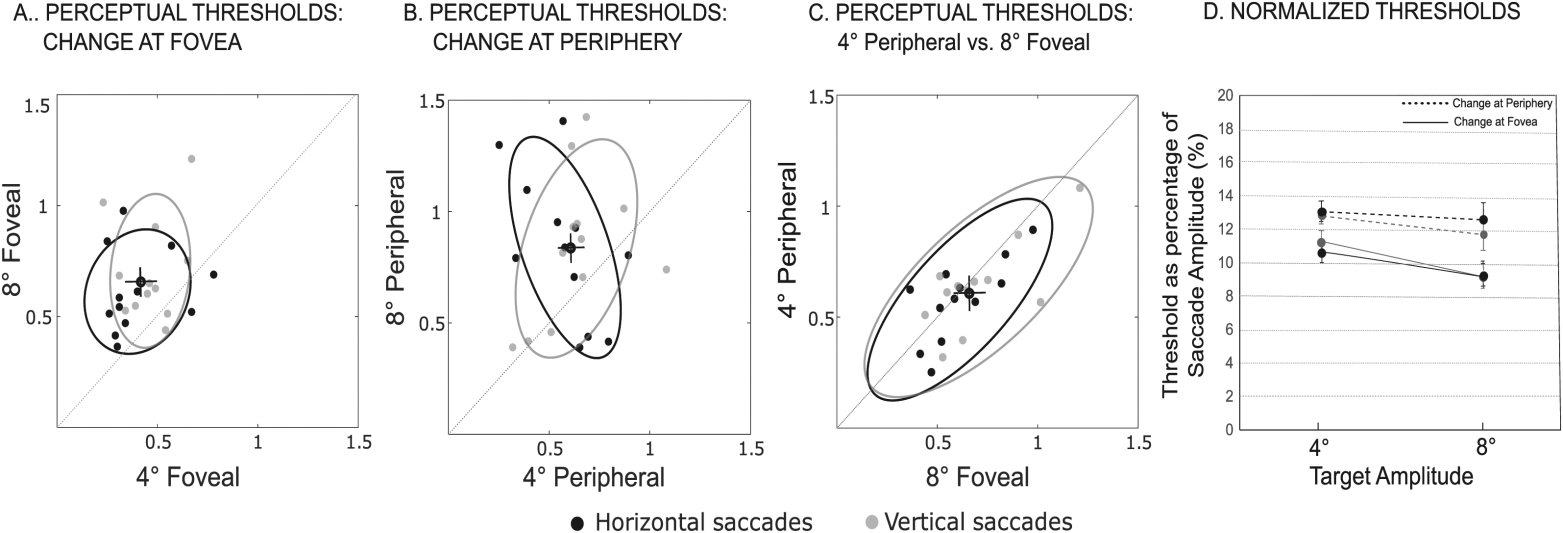
Comparison of the perceptual thresholds across trial types. (A-C) In each plot filled circles represent individual subject data for saccades to the different eye-movement target. Gray plots represent those for vertical saccadic targets and black for horizontal targets. The crosshair represents the respective mean thresholds for both conditions and ellipses represent 95% confidence intervals. (A) Foveal perceptual thresholds for 8° saccadic eye-movement target (and reference point), plotted as a function of the respective threshold for 4° saccadic eye-movement target. (B) Trials in which environmental changes were in the periphery: Perceptual thresholds for 8° saccadic eye-movement targets (with reference point at 12°), plotted as a function of the respective threshold for 4° saccadic eye-movement (with reference point at 8v). (C) 4° Peripheral 8° foveal thresholds: Perceptual thresholds for trials in which the saccadic eye-movement target was at 4°, but shift was at 8° as a function of perceptual thresholds for trials in which the saccadic eye-movement target and shift were both at 8°. (D) Comparison of normalized perceptual thresholds: Average normalized (with respect to saccade length) thresholds for all trial types. Dashed lines represent data for foveal shifts, solid lines depict results for peripheral shifts; Gray for vertical eye movements, and black for horizontal eye movements. Respective vertical lines represent the SE across subjects.

The perceptual threshold results across all subjects for each condition are summarized in Figure 3. Figure 3A displays the summary data for trials in which the environmental change occurred at the fovea (at the 4° and 8° targets), whereas 3B shows the summary data for trials in which the environmental change occurred in the periphery (4° away from the 4° and 8° targets). For changes at the fovea (Figure 3A), threshold measurements were significantly greater for saccades to the 8° saccadic eye-movement targets compared to saccades to the 4° saccadic eye-movement targets, but the thresholds were not significantly different between the two saccade directions (two-way ANOVA, F_1,11_ = 6.67, *p* = 0.025, η^2^_p_ = 0.38 for the main effect of saccade amplitude and F_1,11_ = 1.32, *p* = 0.27, η^2^_p_ = 0.11 for the main effect of saccade direction). We did not observe a significant Direction and Amplitude interaction effect (F_1,11_ = 0.07, *p* = 0.80, η^2^_p_ = 0.01). Similarly, for trials in which environmental changes were in the periphery (Figure 3B), perceptual thresholds were significantly greater for saccades to the 8° saccadic eye-movement targets compared to saccades to the 4° saccadic eye-movement targets, but not significantly different between the two saccade directions (two-way ANOVA, F_1,11_ = 17.61, *p* = 0.001, η^2^_p_ = 0.62 for the main effect of saccade amplitude and F_1,11_ = 0.25, *p* = 0.63, η^2^_p_ = 0.02 for the main effect of saccade direction) and a non-significant Direction and Amplitude interaction effect (F_1,11_ = 0.16, *p* = 0.70, η^2^_p_ = 0.01). Thus similar to the sample subject, the difficulty in shift detection increased with saccade amplitude across all trial types (horizontal and vertical saccades, and the environmental change at the fovea and in the periphery).

Next we examined the effect of shift location (foveal versus peripheral) on the perceptual threshold. We investigated the extent, for reference shifts occurring at 8°, making an eye movement to the 4° saccadic eye-movement target still confers a movement-related perceptual benefit to detecting shifts in the periphery. To do so, for both movement directions, we compared perceptual thresholds for trials in which saccades were made to the 4° saccadic eye-movement target (with the shift occurring 4° away at the 8° location) to trials in which the saccades and reference shift were both at the 8° location. In a two-way ANOVA, we found no main effect of shift location (F_1,11_ = 0.78, *p* = 0.40, η^2^_p_ = 0.07) and no main effect of direction (F_1,11_ = 2.03, *p* = 0.18, η^2^_p_ = 0.16). As indicated by these results and as shown in Figure 3C (where the data is aligned with the unity line: horizontal movements *r* = 0.73, *p* = 0.007; vertical movements, *r* = 0.70, *p* = 0.01) the difficulty in making perceptual judgments about transsaccadic shifts was roughly equivalent, suggesting that the ability to detect the change in the periphery following a small amplitude saccade(4° saccade, 8° reference point) was similar to performance for a larger saccade made close to the location of the environmental change (8° saccade, 8° reference point). It is known that the perceptual threshold increases with movement amplitude and we observe that thresholds are greater for peripheral changes compared to foveal. By examining the case where the change is in the same location, but the saccade amplitudes are different (foveal vs. peripheral) we determined where these effects on threshold are similar.

Furthermore, to determine the extent there was a systematic relationship between saccade amplitude and threshold, we compared the normalized thresholds (Figure 3D). For each subject, we scaled each threshold by that subject's mean saccade amplitude, in this case defining the threshold as a percentage of the movement length. We observed that these normalized thresholds were approximately constant across all movement directions and amplitudes, but larger for trials in which trans-saccadic changes were in the periphery. This was supported by a three-way ANOVA in which we observed a significant main effect of reference shift location (F_1,11_ = 37.01, *p* < 0.001, η^2^_p_ = 0.77). As displayed in Figure 3D, the normalized threshold was slightly higher for 4° versus 8° saccades, but this did not approach statistical significance (F_1,11_ = 3.82, *p* = 0.08, η^2^_p_ = 0.26 for the main effect of saccade amplitude). Normalized thresholds were not significantly different across saccade direction (F_1,11_ = 1.97, *p* = 0.19, η^2^_p_ = 0.15, for the main effect of saccade direction). Neither the three-way location, direction, and amplitude interaction effect (F_1,11_ < 0.001, *p* = 0.98, η^2^_p_ = 0.00), nor any of the two-way interaction effects (location and direction [F_1,11_ = 0.50, *p* = 0.50, η^2^_p_ = 0.04]; location and amplitude [F_1,11_ = 0.95, *p* = 0.35, η^2^_p_ = 0.08]; direction and amplitude [F_1,11_ = 0.28, *p* = 0.61, η^2^_p_ = 0.03]) were significant.

For foveal shifts, consistent with previous studies, the normalized threshold was approximately 10% of the movement amplitude for all directions and amplitudes tested (overall foveal normalized threshold, mean of 10.01%, SEM = 0.44). Comparatively, for peripheral shifts, the normalized threshold was a higher percentage of the movement amplitude across directions and target amplitude (overall peripheral normalized threshold, mean of 12.48%, SEM = 0.57), leading to a significant difference between normalized thresholds for peripheral versus foveal shifts; paired *t*-test, t(11) = 6.08, *p* < 0.001, Cohen's d = 1.76.

### Perceptual bias and percent error of reference point location

To examine the relationship between the perceptual bias, saccade metrics and reference point shift location, we compared the percent error in the reference point location and the percent gain of the saccade amplitude. A three-way ANOVA on the perceptual bias revealed a significant main effect of reference shift location (F_1,11_ = 5.69, *p* = 0.04, η^2^_p_ = 0.34) and amplitude (F_1,11_ = 8.82, *p* = 0.01, η^2^_p_ = 0.45 for the main effect of saccade amplitude). Perceptual biases were not significantly different across saccade direction (F_1,11_ = 0.13, *p* = 0.73, η^2^_p_ = 0.01, for the main effect of saccade direction). Neither the three-way location, direction, and amplitude interaction effect (F_1,11_ = 0.87, *p* = 0.37, η^2^_p_ = 0.07), nor any of the two-way interaction effects (location and direction [F_1,11_ = 0.05, *p* = 0.83, η^2^_p_ = 0.00]; location and amplitude [F_1,11_ = 2.35, *p* = 0.15, η^2^_p_ = 0.18]; direction and amplitude [F_1,11_ = 0.01, *p* = 0.92, η^2^_p_ = 0.00]) were significant.

**Figure 4.**
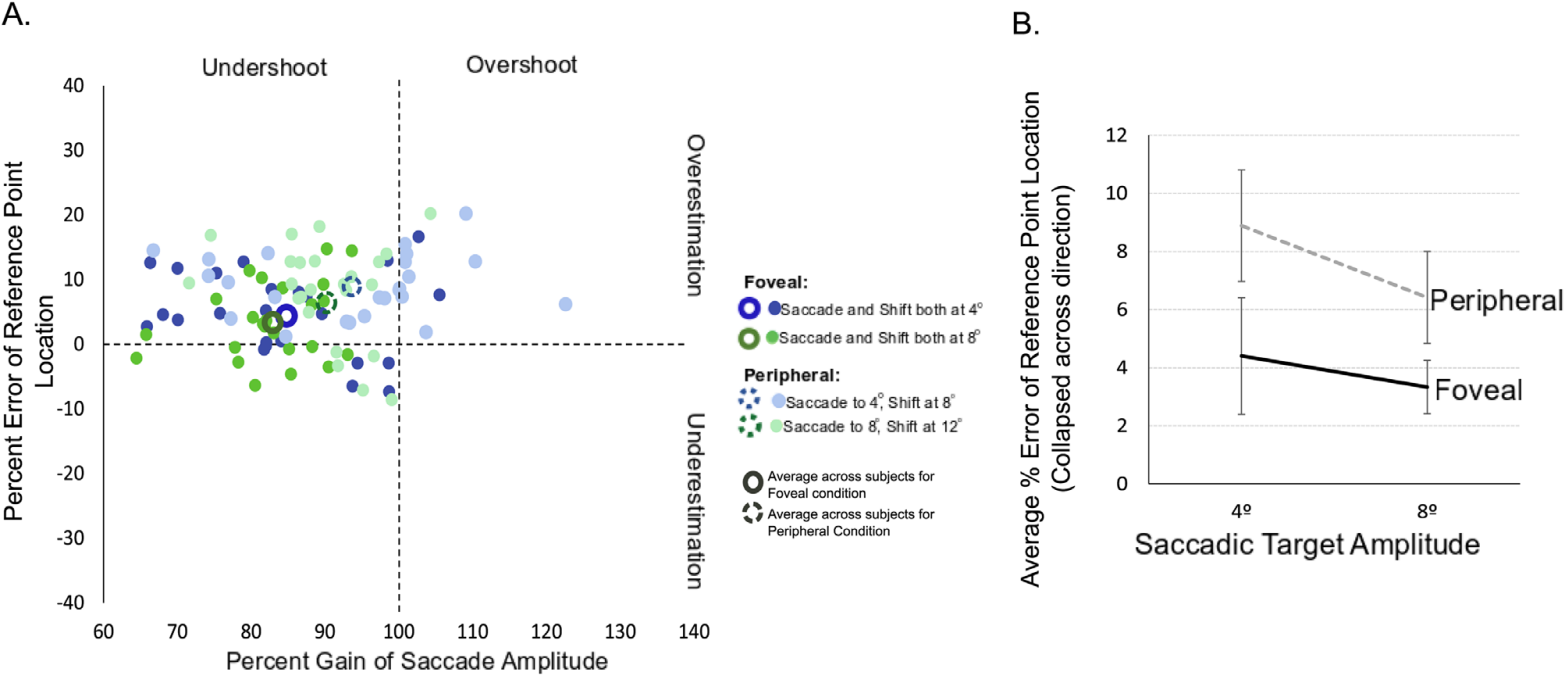
(A) Percent gain of saccade amplitude and percent error estimation of reference point location. The percent error in the estimated reference point location is plotted as a function of percent gain in the saccade amplitude for all saccade amplitudes and directions, separated by foveal and peripheral conditions. Horizontal and vertical movements are combined for each amplitude, and the larger unfilled symbols represent the respective mean percent gains in saccade amplitude and mean percent errors in target location estimation (blue: 4° saccadic eye-movement target, and green: 8° saccadic eye-movement target; lighter shading: reference location shift in the periphery; darker shading: foveal shift of reference location). (B) Mean percent errors of reference point location. The error in reference point location is shown for the two saccadic eye-movement target amplitudes for each environmental change (foveal and peripheral) collapsed across movement direction.

In Figure 4A, we plot the percent error of the estimated reference point location (see Methods, >0 overestimation, <0 underestimation) as a function of the percent gain of the mean saccade amplitude (>100% overshoot, <100% undershoot) for all saccade amplitudes and directions, and reference point shift locations. The former is the perceptual bias as a percentage of the reference point amplitude. The latter is the saccade amplitude as a percentage of the required movement amplitude.

The mean percent error of the estimated reference point location was significantly greater than 0 for all saccade directions, amplitudes and reference point shift locations (*p* < 0.05 for all 8 comparisons, one sample two-tailed *t*-test) but this percentage was not different between horizontal and vertical saccades for each amplitude and shift location (*p* > 0.08 for all four comparisons, paired *t*-tests). Thus we combined the horizontal and vertical movements to specifically examine differences across movement amplitude and reference point shift location. As shown in Figure 4A, despite regularly making movements less than the required target amplitude, subjects largely (81 of 96 cases, 84.4%) overestimated the reference point location (mean percent errors, collapsed across direction are shown in Figure 4B). Considering that the saccadic target and reference point onset and offset were simultaneous, there may have been temporal compression effects; however, compression of the peripheral probe would have led to an inward shifted perception of the presaccadic probe resulting in a more negative judgement bias, whereas we see here that subjects largely overestimated the reference point location (largely a positive bias).

The percent error of the estimated reference point location was significantly larger for peripheral shift trials than for trials in which the shift was at the fovea, but the percent error of reference point location was not significantly different across the two saccade amplitudes (two-way ANOVA, main effect of shift location, F_1,11_ = 5.07, *p* = 0.046, η^2^_p_ = 0.32, main effect of saccade amplitude, F_1,11_ = 3.29, *p* = 0.097, η^2^_p_ = 0.23), with no significant location and amplitude interaction effect (F_1,11_ = 0.21, *p* = 0.66, η^2^_p_ = 0.02). Thus the difference between the perceptual reference point location estimate (i.e., the perceptual bias) and the actual reference point location were greater when judgements about trans-saccadic shift directions were made for a peripheral change compared to at the fovea.

### The benefit of extraretinal information: Postsaccadic estimation of the presaccadic reference point location

**Figure 5.**
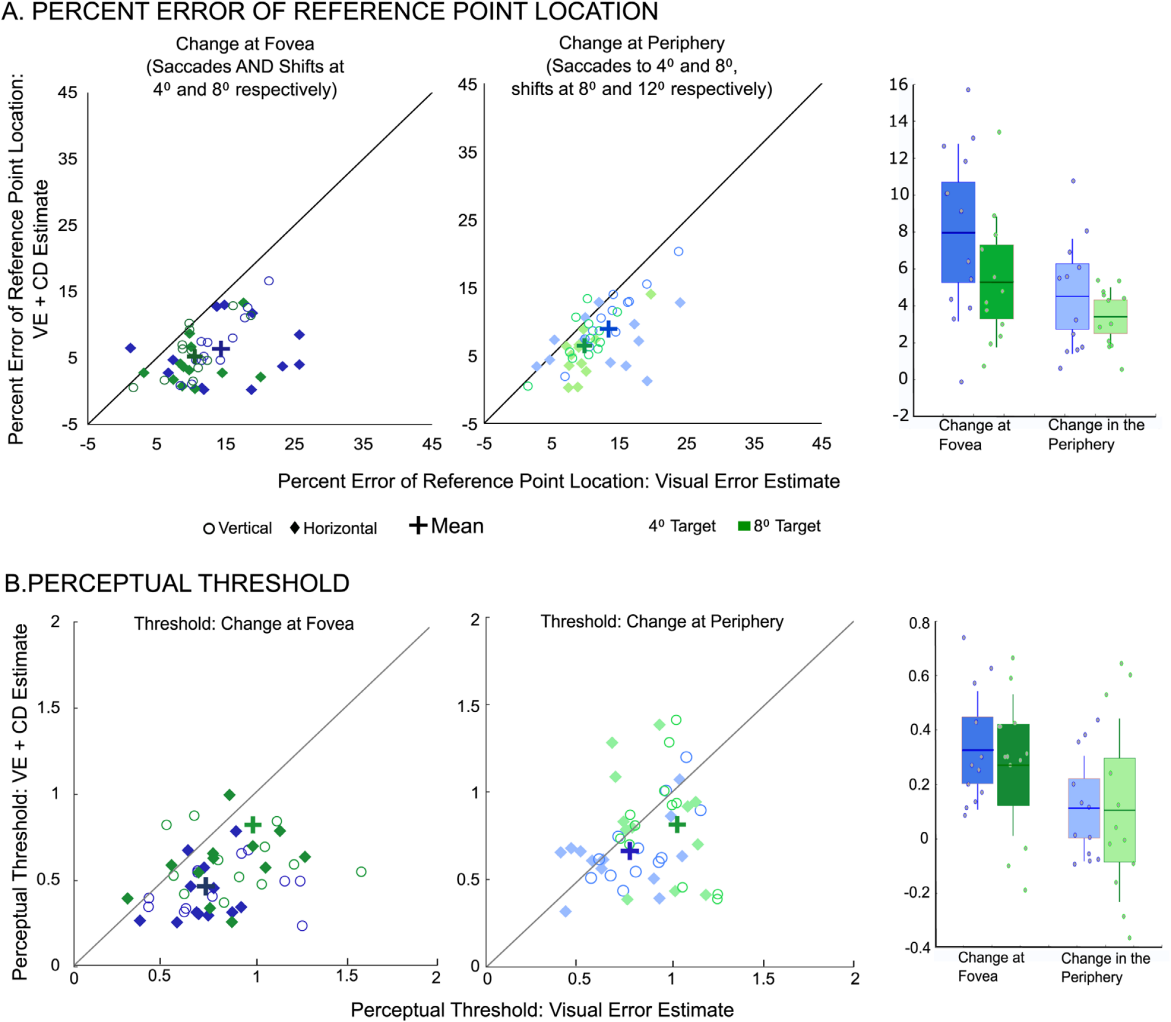
(A) Comparison of the post-saccadic reference point location estimation based solely on post-saccade visual information (VE) or with additional extraretinal information(VE + CD). Panel 1 and 2: Individual data points (circles and diamonds) and average (bold cross symbols) % errors of target location (Visual Error + Extraretinal- vs. Visual Error-based estimates) are plotted for each target amplitude (blue: 4° saccadic eye-movement target, and green: 8° saccadic eye-movement target) and direction (unfilled circles: horizontal, and filled diamonds: vertical). The first panel displays these data for changes at the fovea and the second (middle) panel for shifts in the periphery. The third panel compares the average respective Visual Error + Extraretinal-based (VE+ CD) and Visual Error-based (VE) measures across subjects. To directly compare the benefit of the eye-movement CD on visual perception near the fovea versus periphery, we derived difference scores between VE + CD based and VE-based percent errors. To simplify this comparison, we collapsed across direction since no significant differences were observed between percent errors for upward and rightward saccades. The third panel displays the average difference scores across subjects. Individual data points are layered over a 1.96 SEM (95% confidence interval) in the box and a 1 SD line. (B) Comparison of the perceptual threshold based solely on post-saccade visual information(VE-based) or with additional extraretinal information (VE+CD). Individual data points and average (bold cross symbols) perceptual thresholds (VE+ CD- vs. VE-based estimates) are plotted for each amplitude and direction, with panel one for changes at the fovea and panel two for peripheral changes. The symbols are the same as in panel A, and the third panel represents difference scores for threshold collapsed across direction.

As in previous studies (Jayet-Bray et al., 2016; Bansal et al., 2018), we were interested in quantifying the extent to which extraretinal information assists accurate visual perception (postsaccadic estimation of the presaccadic reference point location) and how the benefit of extraretinal information is affected by the saccade metrics (amplitude and direction) and reference point shift location (foveal versus peripheral). To directly compare the accuracy and confidence of the combined post-saccade visual error (probe visual error) and extraretinal (CD) signal-based perceptual performance (VE + CD for short) to that based solely on the available post-saccade visual error information (VE) we determined the psychometric curves when the shift direction judgment was dependent on the post-saccadic visual error experienced at reference point reappearance (see Methods). Based on the subsequent psychometric functions we computed the estimate of the pre-saccadic reference point location (the hypothetical perceptual bias if the report of the shift direction was based solely on the VE). These estimates of the reference point location and confidence of the location estimates were then compared when based only on the postsaccade visual error (VE- based) or on the actual perceptual judgments (VE + CD; Figure 5A). We determined the bias for all eight conditions (two saccade amplitudes, two saccade directions, and two reference point locations) for each subject. For all saccade directions, amplitudes and shift locations, there was no linear relationship between the VE +CD and VE-based perceptual biases (*p* > 0.35 for all cases).

We were interested in the magnitude of the estimation error when only reliant on the VE rather than the direction of the respective errors. The percent error of the target location was thus calculated from the absolute bias measures of the psychometric functions for each subject and summarized in Figure 5. In Figure 5A, the VE + CD percent error in target location is plotted as a function of the corresponding VE-based percent error. First, when considering the condition in which trans-saccadic shifts occurred at the fovea (left panel, Figure 5A), VE-based percent errors were significantly greater than VE + CD based percent errors across both directions and amplitudes (three-way ANOVA, main effect of CD benefit, F_1,11_ = 55.45, *p* < 0.001, η^2^_p_ = 0.83) (Note that the majority of the data and the respective mean values (represented by the crosses) across subjects are below the unity line). Overall, when collapsing across amplitude and direction, this perceptual estimate at the fovea was approximately 53% more accurate (VE + CD mean = 5.80 vs. VE-only mean = 12.43) and 30% less variable (VE + CD SD = 4.29 vs. VE-only SD = 6.16) than estimates based solely on visual information. The magnitude of percent error was lower for 8° saccadic eye-movement targets than for saccades to the 4° saccadic eye-movement targets as evidenced by a main effect of amplitude (F_1,11_ = 4.91, *p* = 0.049, η^2^_p_ = 0.32), but percent error was similar across horizontal and vertical conditions (main effect of saccade direction, F_1,11_ = 0.37, *p* = 0.55, η^2^_p_ = 0.03). None of the two- or three-way interactions were statistically significant (*p* > 0.1 for all cases). Similarly, when considering peripheral shifts, VE-based percent errors were significantly greater than VE + CD based percent errors across both directions and amplitudes (three-way ANOVA, main effect of CD benefit (F_1,11_ = 122.93, *p* < 0.001, η^2^_p_ = 0.92) (As in the left panel, here again the majority of the data and the respective mean values across subjects are below the unity line). For peripheral shifts, when collapsing across amplitude and direction, the perceptual estimate was approximately 34% more accurate (VE + CD Mean = 7.65 vs. VE-only mean = 11.62) and 9.5% less variable (VE + CD SD = 3.98 vs. VE-only SD = 4.41) than estimates based solely on visual information. The magnitude of percent error was significantly lower for 8° saccadic eye-movement targets than for saccades to the 4° saccadic eye-movement targets (main effect of amplitude, F_1,11_ = 8.76, *p* = 0.013, η^2^_p_ = 0.44), and slightly greater for vertical saccadic targets, but this main effect of direction did not reach significance (F_1,11_ = 3.51, *p* = 0.09, η^2^_p_ = 0.24). Here, like for the foveal shifts, none of the two- or three-way interactions were significant (*p* > 0.15 for all cases)

To directly compare the benefit of the eye-movement CD on visual perception near the fovea versus periphery, we derived difference scores between VE + CD based and VE-based percent errors, with a greater difference indicating a greater benefit that eye movement-related CD confers for that particular condition. To simplify this comparison, we collapsed across direction since no significant differences were observed between percent errors for upward and rightward saccades. The third panel in Figure 5A displays the difference scores. In a two-way ANOVA, including amplitude and shift location as main effects, we found that the eye movement related CD benefit (magnitude of difference score) was significantly lower for saccades to the 8° target versus the 4° saccadic eye-movement target across both shift locations (significant main effect of amplitude, F_1,11_ = 6.29, *p* = 0.029, η^2^_p_ = 0.36). We also found a significant main effect of shift location (F_1,11_ = 12.17, *p* = 0.005, η^2^_p_ = 0.52), indicating that eye movement related CD benefits perception more at the fovea than in the periphery. However, the CD benefit in the periphery was significantly greater than 0 for both 4° and 8° targets (one sample t-test, 4° saccadic eye-movement target: t(11) = 5.01, *p* < 0.001, Cohen's d = 1.45; 8° saccadic eye-movement target: t(11) = 7.44, *p* < 0.001, Cohen's d = 2.15), indicating that eye movement related CD still benefited perception in the periphery, although to a lesser degree compared to the fovea.

Finally, we directly measured the extent making an eye movement to the 4° saccadic target still confers an eye movement-related perceptual benefit for environmental shifts occurring at 8°. That is, we compared the difference score for the perceptual bias (between the VE + CD based and the VE-based percent error) for trials in which saccades were made to the 4° saccadic eye-movement target (with the shift occurring 4° away at the 8° location) to trials in which the saccades and shift were both at the 8° location. The difference scores were similar, with no significant difference between them (mean of 5.30, SEM = 1.02° for the foveal shift, mean of 4.52^o^, SEM = 0.90° for the peripheral shift, paired *t*-test t(11) = 0.79, *p* = 0.45, Cohen's d = 0.23). Thus, similar to our threshold results (see Figure 3C), the extent to which CD assists visual perception in the periphery after a small amplitude saccade was similar to the CD benefit conferred for a larger saccade made close to the location of the environmental change.

### The benefit of extraretinal information (CD): Perceptual threshold

In addition to assessing the utilization of CD in terms of perceptual bias, we also examined the precision of perceptual judgments (or sensitivity to reference point shift detection) as quantified by the perceptual threshold. As described above for the perceptual bias, we derived estimates of perceptual threshold based on the hypothetical psychometric curves based solely on the post-saccadic VE and compared these to the actual perceptual thresholds based on the manual responses. In doing so, we not only examine the CD contribution to post-saccadic estimation of the pre-saccadic initial target, but also to the ability to detect post-saccadic shifts in spite of eye movement variability.

As above, we collapsed thresholds across direction for this analysis and compared conditions in a three-way ANOVA, with factors of saccade amplitude, reference point shift location and CD benefit (VE +CD versus VE-only). As shown in Figure 5B, for both foveal and peripheral shift locations, both VE+CD-based and VE-only based thresholds were significantly greater for 8° saccadic eye-movement targets than the 4° targets (means indicated by crosses in both panels), resulting in a significant main effect of movement amplitude (F_1,11_ = 8.01, *p* = 0.016, η^2^_p_ = 0.42). This indicates less precision in the overall detection of the reference point shifts for larger-amplitude saccades, consistent with the results in Figure 3. Trans-saccadic shift perception was more difficult for shifts occurring in the periphery than at the fovea (main effect of shift location, F_1,11_ = 34.59, *p* < 0.001, η^2^_p_ = 0.76). Furthermore, as with percent error above, across both foveal and peripheral conditions, the perceptual thresholds based on probe visual error (overall VE-only mean threshold of 0.84°, SEM = 0.03°) were significantly greater than the actual CD-utilization based perceptual thresholds overall (mean of 0.63, SEM = 0.04°) (main effect of CD benefit, F_1,11_ = 18.47, *p* = 0.001, η^2^_p_ = 0.63). This indicates greater precision in the shift detection when the CD is used, versus an incorrect reliance based solely on the experienced visual error. There was a significant two-way interaction of shift location and CD benefit (F_1,11_ = 5.15, *p* = 0.04, η^2^_p_ = 0.32). This indicates that there was greater precision in the shift detection when the CD is used, versus an incorrect reliance based solely on the experienced visual error, and this greater precision was more so for shifts occurring at the fovea than in the periphery. However, the three-way interaction of saccade amplitude, reference point shift location and CD benefit (F_1,11_ = 0.29, *p* = 0.6, η^2^_p_ = 0.03) and other two-way interactions did not reach significance (*p* > 0.6 for all cases).

As above, we directly measured the extent making an eye movement to the 4° saccadic eye-movement target still conferred an eye movement-related perceptual benefit for detecting environmental shifts occurring at 8°. That is, we compared the difference score for the perceptual threshold (between the VE + CD based and the VE-based threshold) for trials in which saccades were made to the 4° saccadic eye-movement target (with the shift occurring 4° away at the 8° location) to trials in which the saccades and shift were both at the 8v location. The smaller the difference score here, the lower the benefit of the utilization of CD. Here, we observed that for the peripheral case (saccades made to 4°, but shift detection was at 8°) the magnitude of the CD benefit (mean difference score = 0.11^o^, SEM = 0.06^o^) was significantly lower than the foveal case, in which both the saccade, and shift was at 8° (mean difference score = 0.32^o^, SEM = 0.06^o^; t paired *t*-test t(11) = 2.99, *p* = 0.01, Cohen's *d* = 0.86). Thus there was a greater CD benefit to the precision of the perceptual judgments for foveal versus peripheral reference point shifts.

### Manual response reaction time

**Figure 6.**
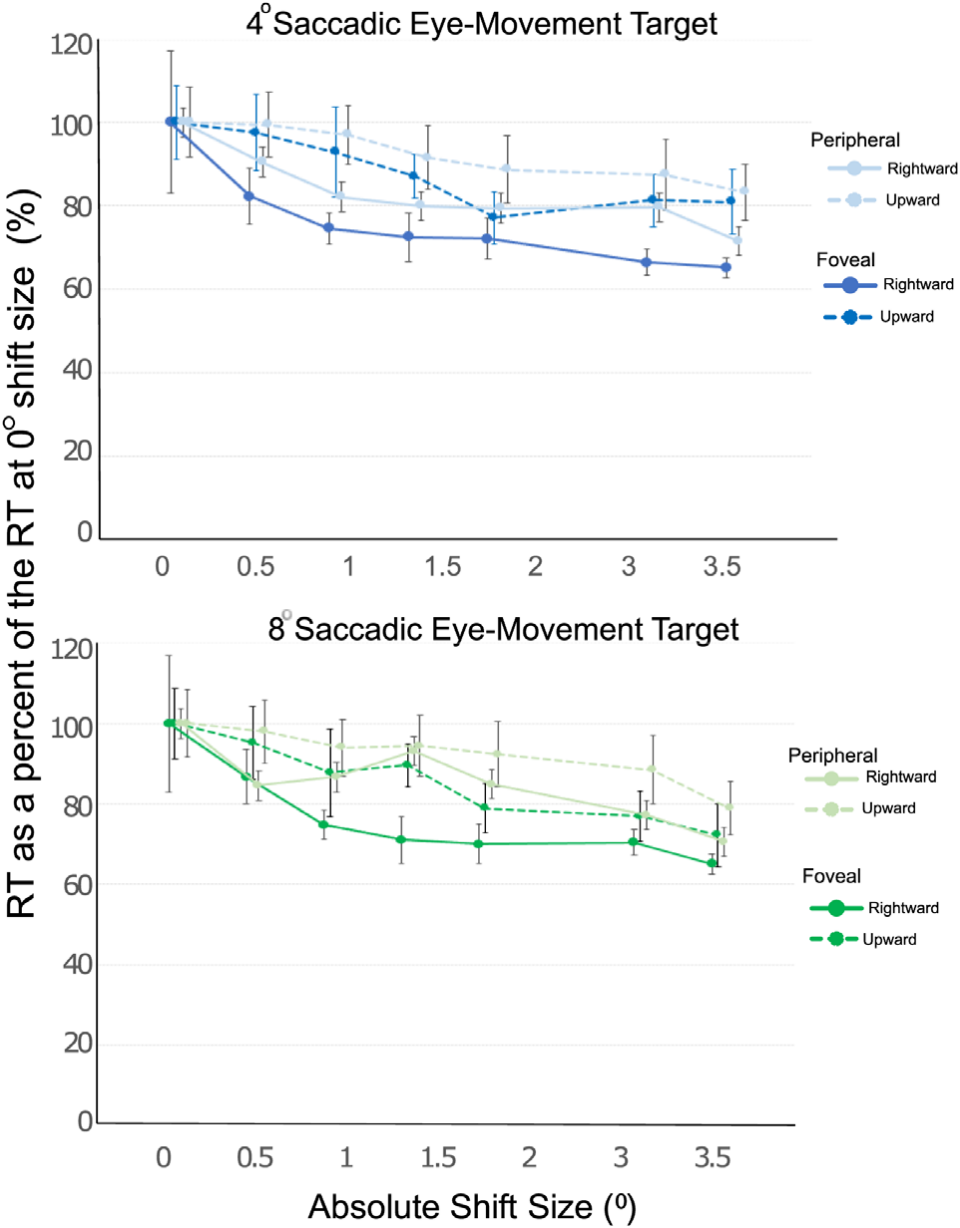
Manual response reaction time. The normalized reaction time (RT) was derived over the absolute shift size. The RTs were normalized by the group mean for each condition so that on the 0° shift the RT was 100% and the RT on the subsequent shift sizes are relative to the 0° shift. Filled circles represent mean values and vertical lines represent standard error. (blue: 4° saccadic eye-movement target, and green: 8° saccadic eye-movement target; lighter shading: reference location shift in the periphery; darker shading: foveal shift of reference location; solid lines: horizontal targets, dashed lines: vertical targets).

In a novel analysis, we examined whether the location of the reference point shift (foveal versus peripheral condition) had an effect on the time required to make a perceptual judgement. We derived manual reaction times per condition, separated by the size of the trans-saccadic shift. (See Figure 6). Here the manual reaction time is derived as the time (in milliseconds) between reappearance of the reference point stimulus at the shifted location and button press time. To compare conditions, we normalized the reaction time (RT); for each condition, we derived the overall mean RT across subjects at the shift of 0° for each condition and then divided all the mean RTs values (for all subjects) by this overall mean RT. This results in a percentage, with the average percentage at the 0° shift across the 12 subjects being 100, with some inter-subject variability. With this method, we were able to observe how much faster (relatively) the perceptual judgments for other shift magnitudes (e.g., >0°) are compared to the most difficult to detect/ambiguous shift size (e.g., 0°). These percentages were collapsed across shift direction (forwards vs. backwards).

We analyzed these normalized RT percentages with an ANOVA with factors of saccade amplitude (4° versus 8°), saccade direction (upwards vs. rightwards), shift location (foveal versus peripheral) and shift magnitude (0.5°-3.5°). First, we observed that normalized RTs overall were longer for perceptual judgments made when the saccadic eye-movement target was at 8° compared to 4°. That is, subjects required a longer RT to make the perceptual judgment for environmental shifts at a larger eccentricity (F_1,11_ = 6.38, *p* = 0.028, η^2^_p_ = 0.37, main effect of amplitude), but RTs for perceptual judgments in the upward versus rightward direction were not significantly different (F_1,11_= 0.70, *p* = 0.42, η^2^_p_ = 0.06, main effect of saccade direction). Importantly, as indicated by the traces in Figure 6A and B, there was a systematic decrease in RTs over shift size, with manual responses being significantly faster when the size of the reference shift was larger (F_1,11_ = 31.22, *p* < 0.001, η^2^_p_ = 0.74, main effect of shift magnitude). Furthermore, there was a main effect of shift location, whereby perceptual judgments at the fovea were made faster than those same shifts in the periphery (F_1,11_ = 37.36, *p* < 0.001, η^2^_p_ = 0.77. main effect of shift location). The four-way interaction (of saccade amplitude and direction, and shift location and size) was not significant (F_5,55_ = 0.76, *p* = 0.53, η^2^_p_ = 0.06). The full description and statistics for all the two- and three-way interaction effects are provided in the appendix. As a comparison (collapsed) across saccadic target amplitude and direction, the overall mean RT for reference location shifts of 3.5° were 29.27% ± 4.7% faster than shifts of 0° at the fovea, while the overall mean RT for reference location shifts of 3.5° were 23.92% ± 4.9% faster than shifts of 0° for shifts in the periphery.

## Discussion

Eye movement characteristics (e.g., direction and amplitude) and the features of visual scene changes in the environment (e.g., near or away from the fovea) combine to influence perception. Understanding their interaction provides insights into the neural mechanisms and strategies that enable visual stability despite the sensory interruptions and variability that accompany the respective eye movements. Previous research has predominantly focused on examining these properties for transsaccadic visual changes occurring at the fovea. Subsequently, there is a gap in understanding how extraretinal information (i.e., corollary discharge) may benefit perception when trans-saccadic displacements occur in the periphery. In the current study we investigated the extent to which extraretinal information supports the ability to perceive trans-saccadic changes at the saccade target location (i.e., near the new fovea location) compared to environmental changes occurring in the periphery (i.e., at a fixed eccentricity away from the saccadic eye-movement target location). We designed a transsaccadic shift detection task that examined the perceptual ability to detect trans-saccadic changes for different movement amplitudes (4° and 8°), directions (upward and rightward), and location of change (at the fovea or in the periphery). We assessed two main characteristics of perceptual performance: the perceptual threshold (the amount of reference point shift at which detection rose sufficiently above chance level) and the perceptual bias (the post-saccadic estimate of the reference point location). As shown previously (e.g. Bansal et al., 2015; Jayet-Bray et al., 2016), we found that perceptual threshold scaled with saccade amplitude for both peripheral and foveal environmental changes with no significant main effect of direction. Second, perceptual thresholds were generally higher for peripheral versus foveal transsaccadic changes and the normalized threshold was a higher percentage of the movement amplitude across target direction and amplitude. However, when directly comparing thresholds for trials in which saccades were made to the 4° saccadic eye-movement target (with the shift occurring 4° away at the 8° location: peripheral change) to trials in which the saccades and reference shift were both at the 8° location (foveal change), we found no significant difference. This suggests that the ability to detect the change in the periphery following a small amplitude saccade was similar to performance for a larger saccade made close to the location of the environmental change. Importantly, we found that the eye movement related CD benefit was greater for 4° saccadic eye-movement targets and, not surprisingly, that eye movement–related CD benefits perception more at the fovea than in the periphery. We quantified this difference by determining a lower difference between CD+VE and VE error-only percent error in the perceptual estimate of the reference location. These collective results suggest a graded use of extraretinal information (CD) when forming the postsaccadic perceptual evaluation of transsaccadic environmental changes and that internal information associated with the saccade size benefits perception (albeit to a lesser degree) even in the periphery.

### Effects of saccade amplitude and change location eccentricity on perceptual threshold

Collectively, the observed systematic changes in perceptual threshold with movement amplitude described above, in agreement with several previous studies in humans (e.g. Bansal et al., 2015; Jayet-Bray et al., 2016; Joiner et al., 2013a; Li & Matin, 1990a; Li & Matin, 1997; Niemeier et al., 2003; Niemeier et al., 2007), may reflect the corresponding neural variability associated with the respective motor commands. The perceptual threshold for foveal reference point shifts is an approximately constant percentage of the movement amplitude, which may reflect the signal-dependent noise previously demonstrated for saccade generation (Goossens & van Opstal, 2012). Here we report that this relationship between movement amplitude and perceptual threshold was also observed for the peripheral shift locations we tested, albeit a higher percentage than that at the fovea.

Previous research (Li & Matin, 1990a; Li & Matin, 1990b) described the displacement threshold as a discernment of a neural signal against a background of neural noise for which a constant signal/noise criterion was assumed. Similar to our results, they found a linear increase of the threshold with an increase of the standard deviation of the psychometric function relating displacement detection to ocular displacement when saccade length was varied systematically (Li & Matin, 1990a; Li & Matin, 1990b). These threshold increases were attributed to saccade-related transient increases in the variability of neural noise related to signals providing extraretinal eye position information (as relayed by corollary discharge) that was involved in stabilizing visual perception of direction against the change in eye position. In later experiments, involving saccades ranging from 4 to 12 degrees in length, Li & Matin (1997) separated the effects of saccade size from the effects of retinal eccentricity of the reference point on saccadic suppression of displacement (SSD). The authors found that the influences of these factors were independent, and that some portion of the basis for the displacement threshold previously ascribed to noise in the extraretinal signal was a consequence of variation in retinal eccentricity of reference and test stimuli. Thus, at least some of the displacement threshold is likely instead to derive from variability in the retinal signal (in the present study, the peripheral eccentricity). That said, Li & Matin (1997) found that 20% of the total influence on SSD was derived from retinal influences of test flash and reference point, but the majority (80%) was due to extraretinal influence associated with saccade size. The current results are consistent with this finding; although the displacement threshold increases with saccade amplitude, it also increases (though less rapidly) when movement size is constant but the retinal eccentricity at which the displacement occurs increases (Figure 3D).

In more recent research, Stewart and Schütz (2019) tested the spatial profile of transsaccadic integration by examining perceptual performance at various locations around the saccade target and between the saccade target and initial fixation. The authors showed that transsaccadic integration, aided by extraretinal signals (e.g., CD) can occur at locations other than the saccade target when the integration location is behaviorally relevant. Coupled with the previous work described above, the collective results show that the extraretinal signals’ benefit to trans-saccadic perception can occur at locations other than the saccadic eye-movement target, but also that the benefit extended to these locations decreases as a function of distance from the saccade goal.

It is important to note that although perception was examined for locations around the saccadic eye-movement target in these previous studies, one main advance of the current work is the ability to directly compare thresholds for foveal and peripheral perception. The results show that perceptual performance for detecting changes in the periphery (retinal eccentricity) following a small amplitude saccade is similar to performance for a larger saccade made close to the location of the environmental change (foveal). Furthermore, examining different movement directions and amplitudes, in addition to the perceptual bias (the post-saccadic estimate of the reference point location), we are able to quantify the benefit of extraretinal information. We derived hypothetical psychometric functions if the perceptual decision was driven by only the post-saccade visual error (VE) between the actual eye location and the shifted reference location. We assume that VE represents the shifted reference location direction, and that the extraretinal information is not utilized to determine the environmental change. This is a simplification, but it provides a baseline under experimental conditions to determine how actual perceptual performance in the foveal versus peripheral conditions (using extraretinal information, specifically CD) is superior to the limited VE-based situation.

With regards to the benefit of movement-driven extraretinal information, it has been found that masking in a displacement task during fixation produced similar results for detection performance as those obtained during saccades (Zimmermann et al., 2014), indicating that the perception benefit is not entirely due to movement. However, when a saccadic eye movement is made, any error/variability due to the saccade has to be taken into account when evaluating the trans-saccadic visual change. In the simulated VE-based situation, motor execution noise (saccade variability) is included into the hypothetical psychometric functions. In both cases (foveal and peripheral) for the VE-based simulation, we assume that subjects believe they made an accurate saccade to the target. Thus, any error they experience is due to the environment. In the peripheral case we assume the subject believes they made a perfect saccade to the 4 (8) degree target. Because the probe is at 8° (12°), a visual error < 4° means the target moved backwards, and a visual error > 4° means the target moved forward. The comparison to the actual psychometric functions (VE+CD) shows that subjects are able to cancel out these self-generated sources of error. That is, there is likely some internal representation of this motor variability in order for it not to corrupt the perceptual decision.

### Neural mechanisms underlying the CD benefit to visual perception at the fovea and periphery

Our behavioral results may relate to the subsequent properties of neural mechanisms that rely on the saccade CD. For example, the experiments of Sommer and Wurtz (2004a, 2004b, 2006) established that the saccade CD plays a role in the anticipatory visual sensitivity demonstrated by frontal eye field (FEF) neurons. Some FEF neurons that receive CD information of the impending eye movement respond to a peripheral visual stimulus before a saccade brings that visual stimulus into their receptive field (Crapse & Sommer, 2012; Joiner et al., 2011; Joiner et al., 2013b; Shin & Sommer, 2012; Sommer & Wurtz, 2004a; Sommer & Wurtz, 2004b; Sommer & Wurtz, 2006; Umeno & Goldberg, 1997). Thus the visual system predicts upcoming input across saccadic eye-movements based on the peripheral preview of the new foveal location. During this retinotopic remapping, neural areas involved in attention and saccade control are activated by a stimulus far outside the respective receptive fields if an impending saccade will bring that stimulus into the classical receptive field location, thus supporting stable trans-saccadic perception (Bays & Husain, 2007; Cavanaugh et al., 2010; Burr & Morrone, 2011; Burr & Morrone, 2012; Higgins & Rayner, 2015; Bisley, Mirpour, & Alkan, 2020).

Presaccadic attention has also been linked to the remapping process (Rolfs, 2015), and it has been suggested that attention creates a retinotopically organized map of both target locations and features at the upcoming saccade location, which is then used to determine which locations are remapped (for review see Cavanagh, Hunt, Afraz, & Rolfs, 2010; Mathôt & Theeuwes, 2011). There is evidence that receptive fields from locations that are attended before a saccade are then remapped, from both neurophysiological (Gottlieb, Kusunoki, & Goldberg, 1998) and behavioral studies (Melcher, 2009). Additionally, the locus of attentional facilitation can be remapped across saccades: studies have shown that attention can be allocated to both the original locus of attention before a saccade, and the retinotopic equivalent of this cued location after the saccade (Golomb, Chun, & Mazer, 2008, Mathôt & Theeuwes, 2010, see also Zirnsak, Lappe & Hamker (2010) for a computational model of perisaccadic perception that unifies presaccadic updating and spatial attention). Thus, in our experiment, even though there is a distinction between the saccadic eye-movement target location and the reference point, the extraretinal CD signal accompanying the saccade could facilitate remapping of not only the saccadic target, but the covertly attended-to reference point location. This would in turn aide trans-saccadic perception of changes occurring in the periphery. However, as seen with our results and others (Li & Matin, 1997), this benefit decreases as the attended-to reference point is located further away from the saccadic eye-movement target.

Further, higher level processes such as visual working memory and attention have been implicated as mechanisms underlying trans-saccadic integration (e.g., Stewart & Schütz, 2018; Stewart & Schütz, 2019), plausibly because of the guidance of attentional pointers in the visual field (Cavanagh et al., 2010). There are disparate findings on the spatial specificity of presaccadic attention, with some studies claiming that attention is linked to the saccade target (e.g., Deubel & Schneider, 1996; Hoffman & Subramaniam, 1995) and others demonstrating that attention may also proliferate to locations around the saccade target (e.g., Harrison, Mattingley, & Remington, 2012; Stewart & Ma-Wyatt, 2017). Attention can also be allocated to a location other than the saccade target during a saccade, when the alternate location is task relevant (White, Rolfs, & Carrasco, 2013; Yao, Ketkar, Treue, & Krishna, 2016). However, given that there is divergent evidence on the spatial profile and specificity of related trans-saccadic processes (e.g., remapping, attention, and memory) it is important to determine the extent of integration benefits at locations other than the saccade goal, and relatedly, for features other than saccade location (e.g., Wolf & Schütz, 2015).

### Reaction time in relation to the perception of visual scene changes

It is important to note that in addition to the perceptual measures of threshold and estimates of the reference location, we also examined the impact of saccade amplitude, trans-saccadic shift size and the shift location on manual reaction times. We found that (1) subjects required a longer RT to make the perceptual judgment for environmental shifts at a larger eccentricity, (2) there was a systematic decrease in RTs over increasing reference location shift size and (3) the perceptual judgments at the fovea were made significantly faster than those same shifts in the periphery. In a recent study, Huber-Huber, Buonocore, Dimigen, Hickey, and Melcher (2019) used concurrent electroencephalography (EEG) and eye-tracking with face stimuli to demonstrate that visual processing and eventual perceptual judgments involve three temporal stages: prediction about the saccadic eye-movement target, integration of presaccadic and postsaccadic information after fixation onset, and postsaccadic facilitation of rapid categorization. These results suggest the existence of fast, pre-working memory integration mechanisms, followed by integration at a working memory stage. Considering these three processes occurring at different stages, it would be expected that factors that influence transsaccadic perception (eccentricity, shift size and shift location) would affect the overall manual reaction time when making perceptual judgements based on the final post-saccadic facilitation of rapid categorization. Thus, as the perceptual judgement difficulty increased with varying saccadic eye-movement target eccentricity and associated noise, as well as with shift size and location, these factors would be expected to result in increased RTs. In a previous study examining reaction times during visual motion perception, Smeets & Brenner (1994) found that when subjects were instructed to react to motion onset, their reaction times were fastest when the target position changed to an extent that resulted in a larger retinal displacement than would arise from the natural variability in the direction of gaze. This is similar to our results in the sense that reference point location shifts occurring at the fovea and larger trans-saccadic shift sizes were discerned faster as compared to reference point location shifts in the periphery and smaller shift sizes. In other words, just as greater velocity of background motion relative to a target decreased manual reaction times, in our study, a greater visual displacement of the target resulted in lower reaction times, and this effect is more pronounced for foveal targets.

## Conclusion

Transsaccadic visual perception and integration has predominantly been tested at the saccade target. In the current study we provide insight on transsaccadic perception and the benefit of extraretinal information to integration of peripheral and foveal information across saccades. In addition, we also provide novel results on perceptual judgement reaction times, showing that similar factors govern both the transsaccadic perception (bias and threshold) and subsequent sensorimotor response associations (reaction time). Based on the presented results, it would be informative to examine how factors like perceptual difficulty (driven by eccentricity and location-related signal-to-noise ratios), covert attention and sensorimotor metrics are related to manual responses proceeding this transsaccadic perception.

## Supplementary Material

Supplement 1
