# Supplementary material for: Transsaccadic visual perception of foveal compared to peripheral environmental changes
Source: J Vis. 2021 Jun 23;21(6):12. doi: 10.1167/jov.21.6.12 (PMC8237106; doi:10.1167/jov.21.6.12)

**APPENDIX** for:

**Trans-saccadic visual perception of foveal compared to peripheral environmental changes**

Sonia Bansal^1,2^ and Wilsaan M. Joiner^3,4,5^

Department of Neuroscience, George Mason University, Fairfax, VA 22030^1^

Maryland Psychiatric Research Center, Department of Psychiatry, University of Maryland School of Medicine, Baltimore, MD 21224^2^

Department of Bioengineering, George Mason University, Fairfax, VA 22030^3^

Department of Neurobiology, Physiology and Behavior^4^, Department of Neurology^5^, University of California, Davis, Davis, CA 95616

## **Manual Response Reaction Time ANOVA results**

We analyzed these normalized RT percentages with an ANOVA with factors of saccade amplitude (4° versus 8°), saccade direction (upwards vs. rightwards), shift location (foveal versus peripheral) and shift magnitude (0.5°-3.5°).

|  | **F** | **p** | **η² _p_** |
| --- | --- | --- | --- |
| Amplitude | 6.38 | ***0.028*** | 0.37 |
| Direction | 0.70 | 0.42 | 0.06 |
| Shift Location | 37.36 | ***< .001*** | 0.77 |
| Shift Magnitude | 31.22 | ***< .001*** | 0.74 |
| Shift Location X Direction | 18.63 | ***0.001*** | 0.63 |
| Shift Location X Amplitude | 5.93 | ***0.033*** | 0.35 |
| ***^GG^*** Shift Location X Shift Magnitude | 1.35 | 0.277 | 0.11 |
| Direction X Amplitude | 78.47 | ***< .001*** | 0.88 |
| Direction X Shift Magnitude | 1.90 | 0.109 | 0.15 |
| Amplitude X Shift Magnitude | 4.31 | ***0.002*** | 0.28 |
| Shift Location X Direction X Amplitude | 31.10 | ***< .001*** | 0.74 |
| ***^GG^*** Shift Location X Direction X Shift Magnitude | 0.76 | 0.518 | 0.06 |
| Shift Location X Amplitude X Shift Magnitude | 1.28 | 0.285 | 0.10 |
| Direction X Amplitude X Shift Magnitude | 1.76 | 0.137 | 0.14 |
| Shift Location X Direction X Amplitude X Shift Magnitude | 0.76 | 0.583 | 0.07 |

***^GG^*** Mauchly's test of sphericity indicated that the assumption of sphericity was violated, the

the degrees of freedom were corrected for deviance from sphericity;

Values in bold indicate statistical significance at p<0.05

Summary of results:

1. Normalized RTs overall were longer for perceptual judgments made when the saccadic eye-movement target was at 8° compared to 4°: subjects required a longer RT to make the perceptual judgment for environmental shifts at a larger amplitude
2. Across shift size, shift location and amplitude, RTs for perceptual judgments in the upward versus rightward direction were not significantly different
3. There was a systematic decrease in RTs over shift size, with manual responses being significantly faster when the size of the reference shift was larger.
4. Perceptual judgments at the fovea were made faster than those same shifts in the periphery
5. The *difference in RTs* for perceptual judgments in the upward versus rightward direction (across amplitude) appeared to be greater for those at the fovea than peripheral changes, leading to a significant interaction effect of Shift Location X Direction
6. Similarly, the *difference in RTs* for perceptual judgments for foveal versus peripheral changes (across direction) was greater when the saccadic eye-movement target was at 8° compared to 4°, leading to a significant interaction effect of Shift Location X Amplitude
7. Across shift size and shift location, for the 4° targets, RTs for perceptual judgments in the upward direction were slower versus rightward the rightward direction, but this was not significant for the 8° targets, leading to a significant interaction effect of Direction X Amplitude.
8. There was a significant interaction effect of Amplitude by Shift Magnitude, such that participants were quicker to make perceptual judgments for larger shifts versus smaller shifts, and the benefit of a larger shift size was more so for 4° targets compared to 8°.
9. Across shift magnitude, there was a significant 3-way interaction between shift location, direction and amplitude, such that normalized RTs were longer for perceptual judgments in the upward versus rightward direction, more so for 8° targets that were in the periphery versus the fovea

We conducted an additional comparison of reaction time for the four cases to determine for what conditions Direction does matter based on the significant interaction for Shift Location x Direction on reaction time (collapsed over Amplitude and Shift Magnitude):

| **Post Hoc Comparisons - Shift Location ✻ Direction** | | | | | | | | | | | |
| --- | --- | --- | --- | --- | --- | --- | --- | --- | --- | --- | --- |
|  | |  | | **Mean Difference** | | **SE** | | **t** | | **p _tukey_** | |
| F,H |  | F,V |  | 5.78 |  | 1.89 |  | 3.06 |  | 0.03 |  |
|  |  | P,H |  | -4.52 |  | 1.79 |  | -2.52 |  | 0.09 |  |
|  |  | P,V |  | -7.65 |  | 2.16 |  | -3.55 |  | 0.01 |  |
| F,V |  | P,H |  | -10.30 |  | 2.16 |  | -4.77 |  | < .001 |  |
|  |  | P,V |  | -13.43 |  | 1.79 |  | -7.48 |  | < .001 |  |
| P,H |  | P,V |  | -3.13 |  | 1.89 |  | -1.66 |  | 0.37 |  |
|  | | | | | | | | | | | |
| *Note.*  Bonferroni adjusted confidence intervals. | | | | | | | | | | | |
| *FH=Foveal, Horizontal; FV=Foveal, Vertical;PH=Peripheral, Horizontal; PV= Peripheral, Vertical* | | | | | | | | | | | |

The reason we may not observe an overall Direction effect is because the changes in RT could be in different directions (cancelling each other out), but there could be a significant difference if we examine the individual Shift Location x Direction cases.

For completeness we performed the same analysis for the Direction x Amplitude interaction:

| **Post Hoc Comparisons - Direction ✻ Amplitude** | | | | | | | | | | | |
| --- | --- | --- | --- | --- | --- | --- | --- | --- | --- | --- | --- |
|  | |  | | **Mean Difference** | | **SE** | | **t** | | **p _tukey_** | |
| H,4 |  | H,8 |  | -10.49 |  | 2.36 |  | -4.44 |  | 0.00 |  |
|  |  | V,4 |  | -13.41 |  | 2.29 |  | -5.84 |  | < .001 |  |
|  |  | V,8 |  | 5.57 |  | 2.31 |  | 2.41 |  | 0.10 |  |
| H,8 |  | V,4 |  | -2.92 |  | 2.31 |  | -1.26 |  | 0.59 |  |
|  |  | V,8 |  | 16.05 |  | 2.29 |  | 7.00 |  | < .001 |  |
| V,4 |  | V,8 |  | 18.97 |  | 2.36 |  | 8.03 |  | < .001 |  |
|  | | | | | | | | | | | |
| *Note.*  Bonferroni adjusted confidence intervals. | | | | | | | | | | | |
| *H,4== Horizontal, 4°saccade; H,8=Horizontal, 8°saccade; V,4=Vertical, 4°saccade; V,8=Vertical, 8°saccade* | | | | | | | | | | | |
|  | | | | | | | | | | | |

**Corrective Saccades**

In order to examine the relationship between corrective saccades and shift magnitude, we conducted an ANOVA with factors of saccade amplitude (4° versus 8°), shift location (foveal versus peripheral) and shift magnitude (0.5°-3.5°). [collapsing across shift direction (backwards or forward) and saccade direction (horizontal or vertical).

The frequency of corrective saccades was larger for 8° targets, with no shift location or shift magnitude effect reaching significance.

|  | **F** | **p** | **η² _p_** |
| --- | --- | --- | --- |
| Amplitude | 9.68 | **0.01** | 0.49 |
| Shift Location | 3.85 | 0.08 | 0.28 |
| Shift Magnitude | 1.79 | 0.12 | 0.15 |
| Shift Location X Amplitude | 0.0001 | 0.99 | 0.00 |
| Shift Location X Shift Magnitude | 1.66 | 0.15 | 0.14 |
| Amplitude X Shift Magnitude | 2.02 | 0.08 | 0.17 |
| Shift Location X Amplitude X Shift Magnitude | 0.56 | 0.76 | 0.05 |

Further, to examine any potential association between perceptual judgements and the frequency of corrective saccades, we examined correlations between these measures. We collapsed across shift direction and magnitude as well as saccade direction to derive the mean percentage of corrective saccades per condition, and related them to mean perceptual thresholds, collapsed across saccade direction.

Although there appears to be a positive linear relationship between these two measures, none of the correlations were significant.


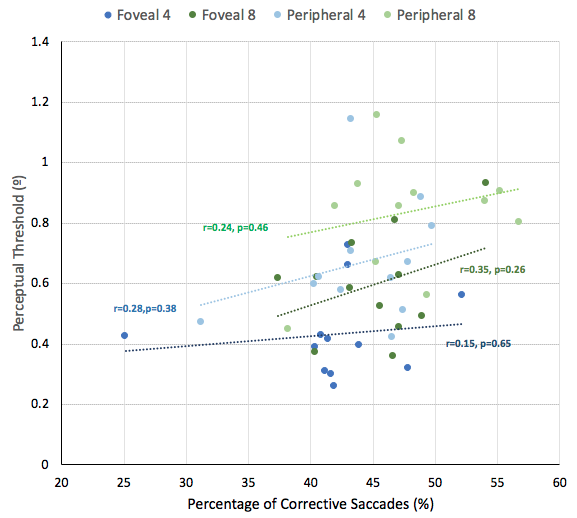

Supplement: Supplement 1 [file jovi-21-6-12_s001.docx]
